# Small Molecule Modulators of AMP-Activated Protein Kinase (AMPK) Activity and Their Potential in Cancer Therapy

**DOI:** 10.1021/acs.jmedchem.4c02354

**Published:** 2025-01-29

**Authors:** Juliet E. Strang, Daniel D. Astridge, Vu T. Nguyen, Philip Reigan

**Affiliations:** † Department of Pharmaceutical Sciences, Skaggs School of Pharmacy and Pharmaceutical Sciences, University of Colorado Anschutz Medical Campus, 12850 East Montview Boulevard, Aurora, Colorado 80045, United States

## Abstract

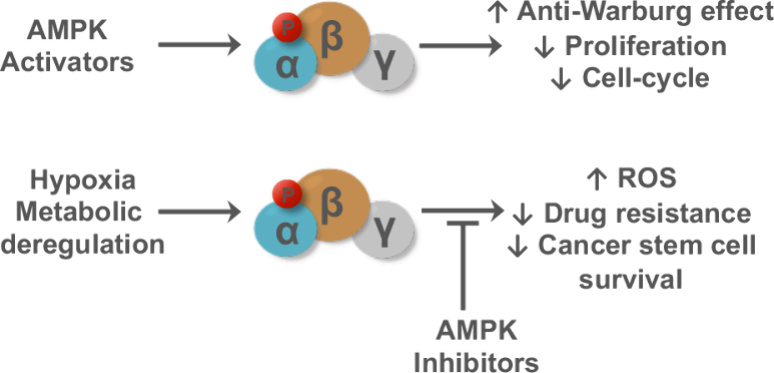

AMP-activated protein kinase (AMPK) is a central mediator of cellular metabolism and is activated in direct response to low ATP levels. Activated AMPK inhibits anabolic pathways and promotes catabolic activities that generate ATP through the phosphorylation of multiple target substrates. AMPK is a therapeutic target for activation in several chronic metabolic diseases, and there is increasing interest in targeting AMPK activity in cancer where it can act as a tumor suppressor or conversely it can support cancer cell survival. Small molecule AMPK activators and inhibitors have demonstrated some success in suppressing cancer growth, survival, and drug resistance in preclinical cancer models. In this perspective, we summarize the role of AMPK in cancer and drug resistance, the influence of the tumor microenvironment on AMPK activity, and AMPK activator and inhibitor development. In addition, we discuss the potential importance of isoform-selective targeting of AMPK and approaches for selective AMPK targeting in cancer.

## Significance

We provide an overview of the role of AMPK in cancer and the development of small molecule modulators of AMPK activity and summarize the indirect and direct AMPK activators and emergent AMPK inhibitors and the challenges of developing these agents as therapeutics. The discussion extends to the importance for tumor-selective AMPK targeting, limitations of cancer models for AMPK modulator evaluation, and the selection of combination therapies, all factors for consideration in future AMPK modulator development for anticancer treatment.

## 1 Introduction

AMP-activated protein kinase (AMPK) is a heterotrimeric serine/threonine kinase that functions as a central metabolic sensor at the interface of metabolic and signaling networks to maintain cellular energy homeostasis.^1−3^ AMPK activation occurs in direct response to low cellular ATP levels as a result of conditions of metabolic stress that cause an increase in the cellular AMP:ATP ratio. Activated AMPK regulates cellular energy by suppressing anabolic pathways that consume ATP and NADPH and promotes catabolic pathways that generate ATP by direct phosphorylation of substrates including metabolic enzymes and transcription factors that influence lipid, cholesterol, carbohydrate, and amino acid metabolism; as well as mitochondrial function and cell growth.^4^ Therefore, it is not surprising that AMPK is a therapeutic target for several metabolic diseases including diabetes, obesity, neurodegenerative and neuromuscular disease, cardiovascular disease, and cancer.^1−3^ In many of these metabolic diseases the objective is to develop small molecules that promote AMPK activity; however, the approach in cancer is more complex due to the dynamic and heterogenic nature of the disease.^5,6^ Furthermore, although there has been intensive research into the development of direct-acting small molecule AMPK activators, few have been evaluated in cancer models. Instead, many studies have used the indirect AMPK activator metformin that have done little to confirm AMPK activation as an anticancer strategy. Similarly, many studies examining the effect of AMPK inhibition have used compound C which has anticancer effects independent of AMPK. However, there have been recent advances in AMPK inhibitor development;^7−9^ therefore, it is timely and important to review the current perspective of AMPK in cancer and the landscape of direct-acting small molecule activators and inhibitors of AMPK.

## 2 Structure and Function of AMPK

### 2.1 Structure of AMPK

**Figure 1:**
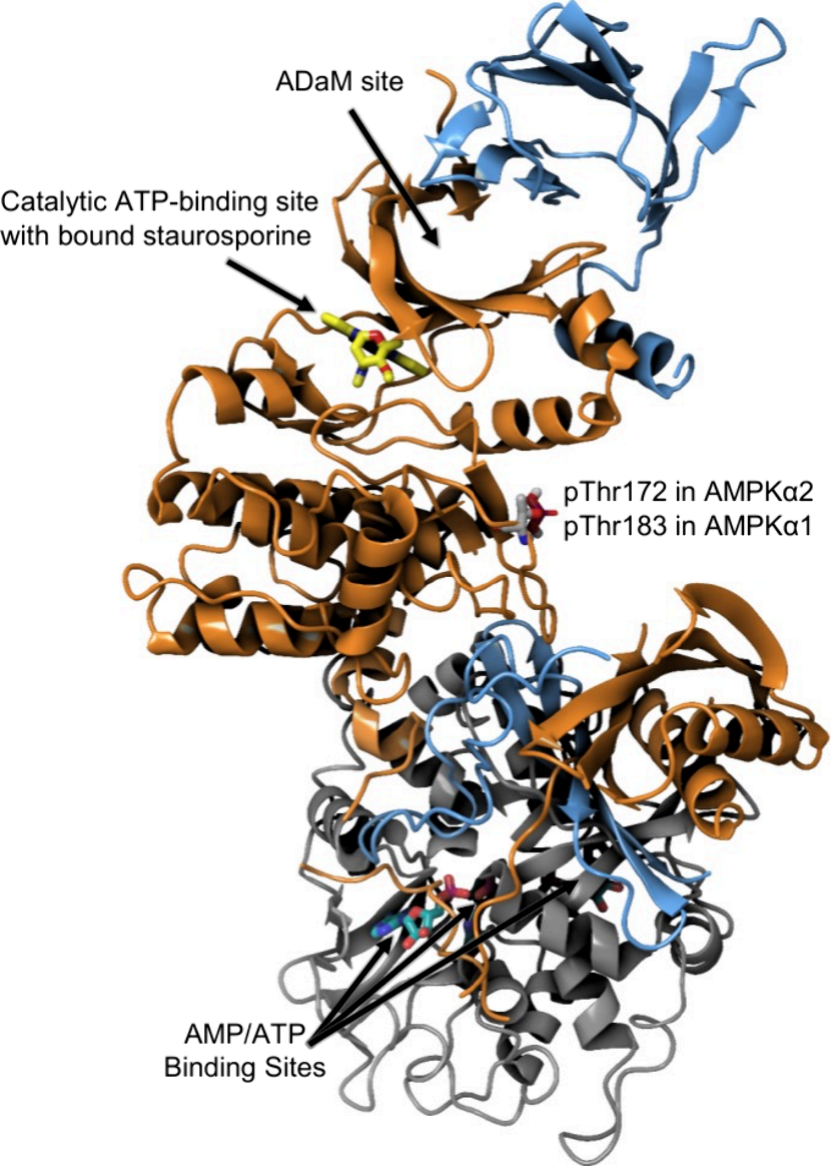
AMPK crystal structure. Ribbon representation of AMPK α2β1γ1 (PDB: 4CFF),^10^ α1-subunit (brown), β2-subunit (blue), γ1-subunit (gray) with phosphorylated Thr residue, the 3 AMP/ATP binding sites, and cocrystallized staurosporine (carbons colored yellow) for reference.

AMPK is composed of three subunits that form a heterotrimeric complex consisting of a catalytic α-subunit (α1 and α2 isoforms), a scaffolding β-subunit (β1 and β2 isoforms), and a regulatory γ-subunit (γ1, γ2, and γ3 isoforms) (Figure 1).^3^

In humans, there are two α-subunit isoforms, α1 and α2, encoded by the *PRKAA1* and *PRKAA2* genes, two β-subunit isoforms, β1 and β2, encoded by *PRKAB1* and *PRKAB2*, and three γ-subunit isoforms, γ1, γ2, and γ3, encoded by *PRKAG1*, *PRKAG2* and *PRKAG3* that have the potential to create 12 distinct heterotrimeric complexes.^11^ These complexes often differ in their tissue distribution, for example the α2 isoform is predominantly expressed in liver, heart, and skeletal muscle,^12^ suggesting that there are specific roles, regulation sensitivities, and different substrate patterns for the isoforms.^13^ The α-subunit contains the Ser/Thr kinase domain (KD) within the N-termini, the conserved Thr172 residue resides on the activation loop of the KD, the KD is followed by an autoinhibitory domain (AID) which is connected to the β-subunit-interacting C-terminal domain (α-CTD) by an α-linker segment.^14^ The β-subunits have an unstructured N-terminus, a glycogen-binding carbohydrate-binding module (β-CBM), and a scaffolding C-terminal domain (β-CTD) that interacts with the α-CTD and the γ-subunit.^14^ The β-CBM forms a cleft with the N-lobe of the α-subunit, above the ATP-binding site of the KD, which serves as a binding site termed the Allosteric Drug and Metabolite (ADaM) site for allosteric activators which will be discussed in a later section. The γ-subunit contains four cystathionine beta-synthase (CBS) motifs forming binding sites for the AMP, ADP, and ATP regulatory nucleotides.^14,15^ One of these binding sites, CBS2, remains unoccupied due to the absence of a conserved Asp residue and the CBS4 site is constantly occupied with AMP. Therefore, the remaining two sites are responsive to changes in the cellular AMP:ATP ratio and the occupancy of these sites initiate the regulation of AMPK kinase activity.^16^

### 2.2 Activation of AMPK

The current canonical model of AMPK activation involves three stages: 1) allosteric activation via AMP/ADP binding to the γ-subunit, 2) phosphorylation of a conserved Thr residue (commonly referred to as Thr172 as numbered in AMPKα2 but Thr183 in AMPKα1), and 3) conformational change to restrict pThr172 dephosphorylation. In conditions where cellular ATP levels are low, AMP or ADP can displace ATP from the regulatory γ-subunit and this initiates AMPK activation through an allosteric mechanism.^16,17^ AMP/ADP binding to the γ-subunit results in a conformational change in the activation loop that exposes a conserved Thr172 residue to phosphorylation primarily by liver kinase B1 (LKB1). The phosphorylation of the Thr172 residue induces further conformational changes in the α-subunit and restricts access of inactivating phosphatases to pThr172 increasing and prolonging AMPK activity.^16,17^ When cells are no longer under energetic stress, AMPK must be inactivated to restore normal cellular metabolic processes. Although dephosphorylation of Thr172 by phosphatases can affect AMPK activity, the primary route for negative regulation is through increased cellular ATP levels that displace AMP in the γ-subunit binding sites. After this exchange occurs, a conformational change occurs that makes AMPK a more efficient substrate for protein phosphatases and Thr172 is dephosphorylated to maintain AMPK in an inactive state.^18^ Several noncanonical AMP/ADP-independent mechanisms of AMPK activation have been reported and include Ca^2+^/calmodulin-dependent protein kinase 2 (CaMKK2), transforming growth factor -β activating kinase 1 (TAK1), lysosomal damage, mitochondrial dysfunction, DNA damage, glucose and glycogen sensing, and fatty acid modulation at the ADaM site and are summarized elsewhere.^16^

### 2.3 Function of AMPK

**Figure 2:**
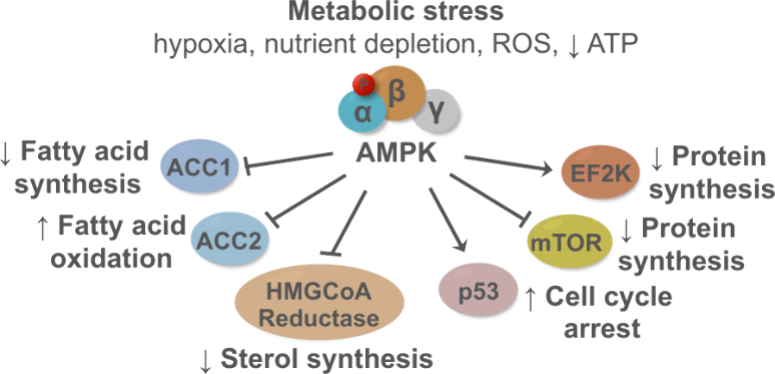
Summary of the phosphorylation targets of AMPK. Metabolic stress induced by hypoxia, nutrient depletion, increased reactive oxygen species, and decreased ATP can activate AMPK to decrease FA synthesis, increase FAO, decrease sterol synthesis, promote cell-cycle arrest, and decrease protein synthesis.

Once activated, AMPK promotes several catabolic processes to generate ATP, including fatty acid uptake and oxidation,^19^ glucose uptake,^20^ glycolysis,^21^ and mitochondrial biogenesis.^22^ Activated AMPK can also conserve cellular energy by suppressing anabolic processes including cell growth and division,^23^ and by inhibiting key biosynthetic processes such as lipid and protein synthesis.^24^ The functional targets of AMPK are summarized in Figure 2.

AMPK directly phosphorylates and inhibits both acetyl coenzyme A carboxylase (ACC) isoforms (ACC1 and ACC2) that mediate the first committed step in fatty acid (FA) synthesis to generate malonyl-CoA.^19^ A reduction in malonyl-CoA indirectly triggers an increase in fatty acid oxidation (FAO) to generate acetyl-CoA. The inhibition of ACC and a reduction in malonyl-CoA results in decrease lipid synthesis and increased FA transportation to the mitochondria for FAO. Several other mitochondrial functions are regulated by AMPK through the regulation of several substrates including mitochondrial fusion by A kinase anchor protein (AKAP1),^25^ mitochondrial fission by mitochondrial fission factor (MFF),^26^ and mitophagy by activation of unc-51-like kinase 1 (ULK1).^27^ The synthesis of cholesterol can be inhibited by AMPK through direct phosphorylation of 3-hydroxy-3-methylglutaryl-CoA reductase (HMGR).^28^ Glucose utilization and uptake into cells can be stimulated by AMPK via phosphorylation of thioredoxin-interacting protein (TXNIP13) and TBC1 domain family member 1 (TBC1D1) increasing the plasma membrane localization of glucose transporters GLUT1 and GLUT4, respectively.^29^ AMPK can also increase flux through the glycolytic pathway by phosphorylating 6-phosphofructo-2-kinase/fructose-2,6-bisphosphatase 3 (PFKFB3), which promotes the activity of phosphofructokinase (PFK1), a rate-limiting enzyme in glycolysis.^30^ Protein synthesis can be inhibited by AMPK via phosphorylation and activation of eukaryotic elongation factor 2 kinase (eEF2K),^31^ and by inactivation of mammalian target of rapamycin complex I (mTORC1) by phosphorylation of tuberous sclerosis complex 2 (TSC2) and mTOR Raptor subunit.^32,33^ AMPK is thought to work in concert with mTOR, a master regulator of cell growth that promotes anabolic pathways under high nutrient conditions, to switch between anabolism and catabolism. AMPK can regulate the cell-cycle via the tumor suppressor p53, which mediates AMPK-dependent G1 cell-cycle arrest, and other key regulators and components of the mitotic machinery to arrest the cell-cycle under low ATP conditions^34−36^

## 3 AMPK in Cancer

The central role of AMPK in metabolically distressed cells has led to its identification as a potential target in cancer. Although increased expression and activation of AMPK has been reported in several cancer types,^6,37^ the precise role that AMPK plays in cancer with respect to functioning as a tumor suppressor or promoter is still a subject of debate. Initial evidence supported that AMPK has a role in tumorigenesis and mediates many of the tumor suppressive effects of LKB1.^38^ Conversely, more recent evidence has supported that the catabolic activity of AMPK may promote tumor growth and survival, and confer drug resistance and resilience under tumor hypoxia.^39−42^ The inconclusive role of AMPK in cancer arises from several key considerations: 1) indirect acting activators, such as metformin, have been used to target AMPK in cancer models which confounds the resolution of AMPK-mediated anticancer activities, 2) the multiple isoforms of AMPK, 3) the heterogenic nature of the disease, and 4) the dynamic nature of tumor growth and the tumor microenvironment (TME), that have been the subject of review but substantial research questions still remain.^5,6^

### 3.1 AMPK as a Cancer Suppressor

**Figure 3:**
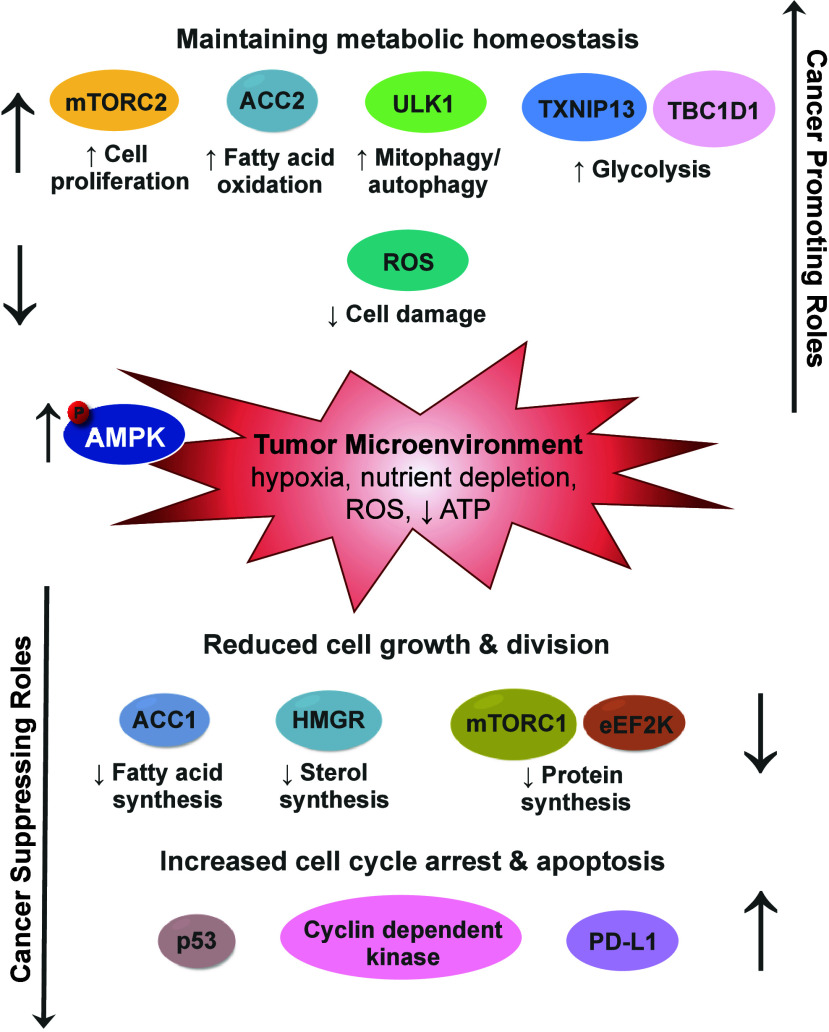
AMPK has a complex role in cancer tumor microenvironments. AMPK activation in cancer is triggered primarily due to metabolic and hypoxic stress. Maintaining metabolic homeostasis is a multifaceted process involving many cell-signaling pathways that are impacted by AMPK activity, and the intricacies of AMPK activity are especially highlighted in the tumor microenvironment where downstream effects are wide reaching and can have contradicting cancer promoting and suppressing effects.

The discovery that AMPK acts downstream of the known tumor suppressor LKB1 and could restrain cell growth via multiple mechanisms supported the assumption that AMPK would also have tumor suppressive actions.^38,43^ The mechanisms by which AMPK could inhibit cell growth and exert a tumor suppressive role include: 1) suppressing FA and cholesterol biosynthesis through direct phosphorylation of ACC1, HMGR, and other substrates;^28^ 2) inhibition of protein synthesis by phosphorylation of mTORC1 and EF2K;^31−33^ and 3) promoting cell-cycle arrest and apoptosis by stabilizing p53 and regulating cyclin dependent kinase (Figure 3).^34,35^

These AMPK-mediated activities, in addition to a potential tumor immunogenic role for AMPK through indirect modulation of programmed cell death ligand 1 (PD-L1),^44^ supported the evaluation of metformin and other AMPK activators as anticancer agents in preclinical cancer models and even in several clinical trials.^2,16^ However, metformin and many of these AMPK activators have AMPK-independent effects; therefore, the anticancer effects observed in these studies may not be directly attributed to increased AMPK activity.

To provide evidence of the role of AMPK in cancer, genetic approaches have been used to determine if loss of AMPK promotes cancer.^45,46^ Although several genetic studies of AMPKα1 knockout mice supported the concept of AMPK as a tumor suppressor, these knockouts were either global (not specific to the tumor progenitor cells) or not all AMPK isoforms were deleted.^45−47^ In hematological cancers the AMPKα1 isoform is predominately expressed; therefore, these cancers have the advantage in that it is only necessary to knockout the *PRKAA1* gene encoding AMPKα1 in lymphomas and leukemia.^48^ The knockout of AMPKα1 in a mouse model of B-cell lymphoma induced by the c-Myc expression accelerated the development of lymphoma, suggesting that AMPK loss allows oncogenic drivers to promote tumorigenesis.^45^ In another study, a knockout of p53 and AMPKβ1, the principal expressed isoform in T-cells, caused an earlier onset of T-cell lymphoma in a mouse model, suggesting AMPKβ1 has a tumor suppressive effect in T-cell lymphoma.^46^ Since these studies, the specific loss of AMPK in tumor progenitor cells has been achieved using a model of T-cell acute lymphoblastic leukemia/lymphoma (T-ALL) and when combined with a phosphatase and tensin homologue (PTEN) knockout, the lymphoma developed at a rapid rate and reduced tumor-free survival.^49^ Collectively, these studies support that AMPK allows oncogenic drivers, such as c-Myc and PTEN loss, to promote more aggressive cancers. In addition, some studies have shown that mTORC1 hyperactivation and subsequent hypoxia inducible factor-1α (HIF-1α) expression in AMPK knockdown cells^45,49^ results in enhanced glycolysis with increased glucose uptake and lactate production, known as the “Warburg effect”, a characteristic trait of cancer cells.^50^ The negative regulation of the Warburg effect by AMPK activation and downregulation of HIF-1α is a central argument to support the role of AMPK as a tumor suppressor.^45^ More recent studies are now focused on the role of AMPK in modulating metabolic plasticity in cancer cells and immune cell types within the TME; however, the role of AMPK may vary across the heterogenetic cancer cell population and may be influenced by the continually adapting TME, as well as the stage and type of cancer.^5^

### 3.2 AMPK as a Cancer Promoter

Conversely, AMPK may protect cancer cells from metabolic stress under nutrient deprivation, hypoxia, or during matrix detachment, thereby promoting tumor survival.^51,52^ Several studies have shown an association between AMPK activation and cancer cell survival, proliferation, and migration, due to the restoration of metabolic homeostasis through increasing catabolic processes and reducing ATP-consuming biosynthetic processes to support cancer cell survival.^53,54^ The main mechanisms by which AMPK could act as a tumor promoter include: 1) the promotion of FAO to generate ATP;^19^ 2) the increase in intracellular NADPH levels to neutralize reactive oxygen species (ROS) via FAO activation and inhibition of FA synthesis;^51^ 3) the activation of mTORC2 that promotes the PI3K-Akt signaling pathway;^55^ and 4) AMPK-mediated autophagy via phosphorylation of ULK1 that confers a metabolic survival advantage in cancer cells and chemoresistance (Figure 3).^27^ AMPK may exert pro-oncogenic activities through direct and indirect regulation of other signaling pathways vital for regulating cell growth and proliferation. AMPK can promote cell-cycle arrest through activation of tumor suppressors such as p53 and p27,^34,35^ which could allow for DNA repair and support drug resistance. Conversely, AMPK inhibition may promote apoptosis via mitotic catastrophe and improve the efficacy of DNA-targeted chemotherapy.^56^

A common view is that AMPK activation may be cancer preventative, perhaps even an effective strategy to target the cancer bulk at certain stages of cancer development, but established cancers or certain heterogenic components under metabolic stress are more sensitive to AMPK inhibition.^16^ The hypoxic activation of AMPK has been shown to be dependent on mitochondrial ROS, an upstream LKB1-independent activator of AMPK, and can also be independent of AMP/ATP ratio.^57^ Under hypoxia, AMPK activation may enhance mitochondrial biogenesis, respiratory capacity, and glucose uptake promoting cancer cell survival.^58,59^ Interestingly, the treatment of lung and colorectal carcinoma cell lines with the direct AMPK activator A769662 has been shown to promote proliferation under hypoxic conditions.^59^ Therefore, while activating AMPK may exert many positive effects with respect to cancer prevention, it will be important that therapeutics targeting AMPK are carefully selected and evaluated in the context of cancer type and stage.

The impact of AMPK loss has been examined in acute myeloid leukemia (AML) and glioblastoma (GBM), and in both these cancer types the expression of AMPK appears to be critical to maintain the viability of the cancer stem cell (CSC) population.^60,61^ This is particularly important as CSCs are quiescent, reside in hypoxic environments, resistant to drugs targeting rapidly dividing cells, and have the ability to initiate tumorigenesis.^62^ In the AML studies, AMPK was required for leukemogenic potential of leukemic stem cells (LSCs), and the deletion of AMPKα1 and AMPKα2 from LSCs in mouse models either delayed the onset of disease or improved survival. Increased ROS levels, reduced NADP and glutathione levels, and increased DNA damage were also observed.^61^ LSCs in AML maintain low levels of ROS and primarily perform oxidative phosphorylation which may be centrally mediated by AMPK. LSCs are sensitive to metabolic changes and disruption of either glycolysis or mitochondrial respiration can prevent leukemogenesis.^63^ Additionally, LSCs tend to reside in the hypoxic niche of the bone marrow where AMPK is activated and AMPK inhibition was shown to sensitize LSCs and suppress AML.^61^ Normal hematopoietic stem cells (HSCs) also reside in this hypoxic environment, but loss of AMPK activity did not affect HSC viability.^61^ Therefore, targeting AMPK activity in LSCs could have important clinical outcomes as LSCs have been found to be involved in disease initiation, progression, and relapse due to their ability to initiate leukemia.^62^ Importantly, recent studies have implicated AMPK in resistance to the FDA-approved Bcl-2 inhibitor venetoclax in leukemia; therefore, AMPK inhibition could be an interesting strategy to potentiate or improve the durability of venetoclax in AML.^64^ In the GBM study, a review of The Cancer Genome Atlas data revealed that the AMPKα1 isoforms were expressed at high levels in GBM, this was confirmed by additional analysis of AMPK isoforms in GBM patient samples compared with normal tissue of low-grade glioma.^60^ Similar to the AML study, high expression of active AMPK was measured in the GBM stem-like cells (GSCs) and knockout of AMPKβ1 decreased the viability of GSCs isolated from patient samples but had little effect on normal astrocytes.^60^ Furthermore, AMPK was reported to phosphorylate cAMP response element binding protein-1 (CREB1) to regulate tumor bioenergetics through HIF1α and nuclear factor erythroid 2-related factor 2 (NRF2), that regulate glycolysis and mitochondria function.^60^

Therefore, there may be a broad cellular context for AMPK-targeted therapeutics as anticancer agents that are effective at different stages of cancer development, for example, small molecules that activate AMPK may be most effective in early stage cancer or in individuals who are predisposed to cancer to prevent tumor initiation/growth.^40^ In contrast, AMPK inhibitors may be most effective for the treatment of established and aggressive cancers or for patients in remission in order to eradicate dormant CSCs and suppress relapse. Although AMPK modulation is a promising therapeutic strategy in several diseases, elucidating its role in cancer requires the development of selective AMPK activators and inhibitors. In the following sections we summarize the advances in indirect and direct small molecule AMPK activators and inhibitors.

## 4 Small Molecule AMPK Activators

The pharmacological activation of AMPK as a treatment strategy for obesity and type-II diabetes was first proposed in the 1990s and led to the development of a diverse range of AMPK activators with different modes of action.^2^ The following section will describe several small molecule AMPK activators categorized by their type of action (indirect/direct) or site of action. These compounds represent some of the most well-known, effective, or recently reported small molecule AMPK activators; however, this is not a comprehensive list of all AMPK activators that have been reported in the literature.

### 4.1 Indirect Activators

The indirect activation of AMPK is in response to compounds that act by increasing the cellular AMP:ATP ratio. The glycolytic inhibitor 2-deoxy-d-glucose (2-DG) can cause rapid AMPK activation in cells that are partially reliant on glycolysis; however, the major generator of ATP is mitochondrial oxidative metabolism and many indirect activators of AMPK target mitochondrial respiration.^2,39^ The biguanide metformin (Table 1), is a widely prescribed oral antidiabetic agent that inhibits complex I in mitochondria, reducing mitochondrial respiration and ATP production and thereby promoting AMPK activation.^65^ The uptake of metformin in the cell is dependent on organic cation transporters expressed at high levels in the liver; however, the lipophilic biguanide phenformin (Table 1) has a greater propensity to enter cells and activate AMPK outside of the liver, but has a risk of lactic acidosis.^66^ Another biguanide derivative, IM156 (HL156A, Table 1), blocks mitochondrial complex I and showed reduced ATP levels in GBM cell lines, but did not activate AMPK suggesting that its anticancer activity is not via an AMPK-dependent pathway.^67^ Astellas reported the discovery of a 3,5-dimethylpyridin4(*H*)-one series as potent indirect AMPK activators, which were further optimized through a medicinal chemistry campaign to generate the benzimidazole ASP4132 (Table 1).^68^ While ASP4132 has been shown to indirectly activate AMPK in cell systems by inhibition of mitochondrial complex I, it may act through multiple other molecular mechanisms to activate AMPK.^69^ Further modification of ASP4132 has resulted in the development of a 3-methylpyridine-based compound 27b (Table 1), with reduced hERG inhibitory activity.^70^

**Table 1:**
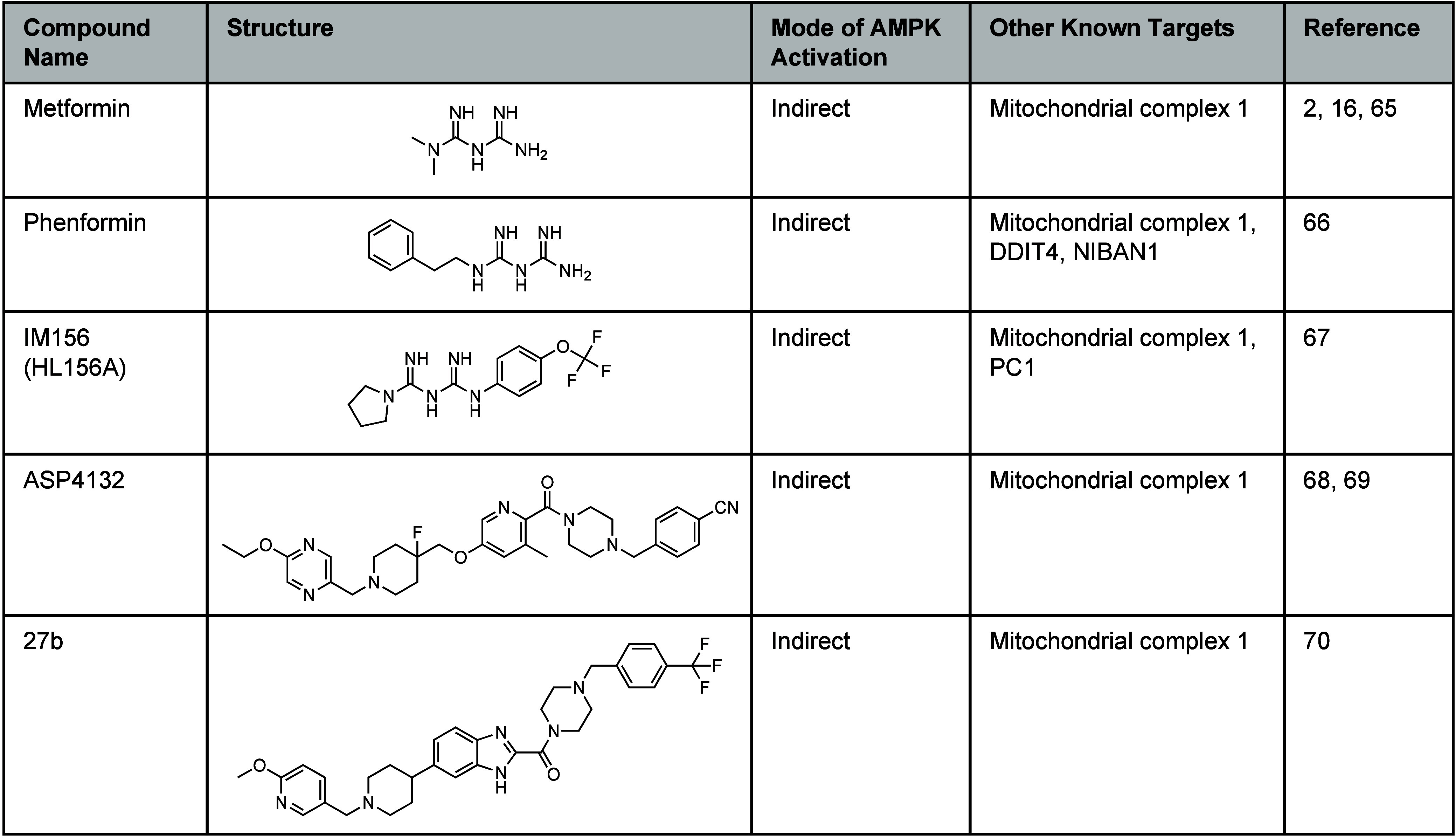
Indirect AMPK Activators^a^

a Chemical structures of metformin, phenformin, IM156, ASP4132, and 27b and a list of known alternative targets.

### 4.2 Adenosine Analogs

The initial efforts to identify direct AMPK activators focused on small molecules that could mimic nucleotide-dependent activation of AMPK at the γ-subunit.^2^ The first direct AMPK activator was 5-aminoimidazole-4-carboxamide ribonucleoside (ZMP, AICA ribonucleotide), the monophosphate derivative of the precursor 5-aminoimidazole-4-carboxamide-1-β-D-ribofuranoside (AICAR, acadesine).^71^ AICAR is an adenosine analog that is taken up into cells by adenosine transporters and phosphorylated by adenosine kinase to the active AMP-mimetic ZMP (Table 2). ZMP binds the AMP-sensing CBS3 site of the γ-subunit of AMPK, resulting in allosteric AMPK activation and protection against Thr172 dephosphorylation.^72^ Although ZMP is less potent than AMP, AICAR can activate AMPK in cells and tissue as it is rapidly converted to ZMP which is not cell permeable and slowly metabolized. Therefore, ZMP can accumulate in the cell and achieve micromolar concentrations necessary for AMPK activation.^71^ The intracellular concentrations of ZMP can give rise to off-target effects with other AMP-sensitive enzymes, including the glycogenolytic enzyme glycogen phosphorylase in cardiac muscle and the gluconeogenic enzyme fructose-1,6-bisphosphatase in the liver.^73^ ZMP is a natural intermediate of purine nucleotide synthesis that is converted to inosine monophosphate (IMP) by AICAR transformylase and inosine monophosphate cyclohydrolase.^74^ The metabolism of ZMP to IMP may explain why AMPK is not activated by AICAR in rapidly proliferating cells that have a high *de novo* nucleotide biosynthetic capacity. AICAR transformylase can be inhibited by folate analogs, such as methotrexate, which are used in the treatment of some cancers and autoinflammatory disorders.^74^ These antifolate drugs inhibit thymidylate synthase and disrupt DNA synthesis, and as a secondary effect this results in inhibition of AICAR transformylase which would allow accumulation of ZMP to promote AMPK activation. However, whether AMPK activation via this mechanism would result in an anticancer effect or support drug resistance is unclear.

**Table 2:**
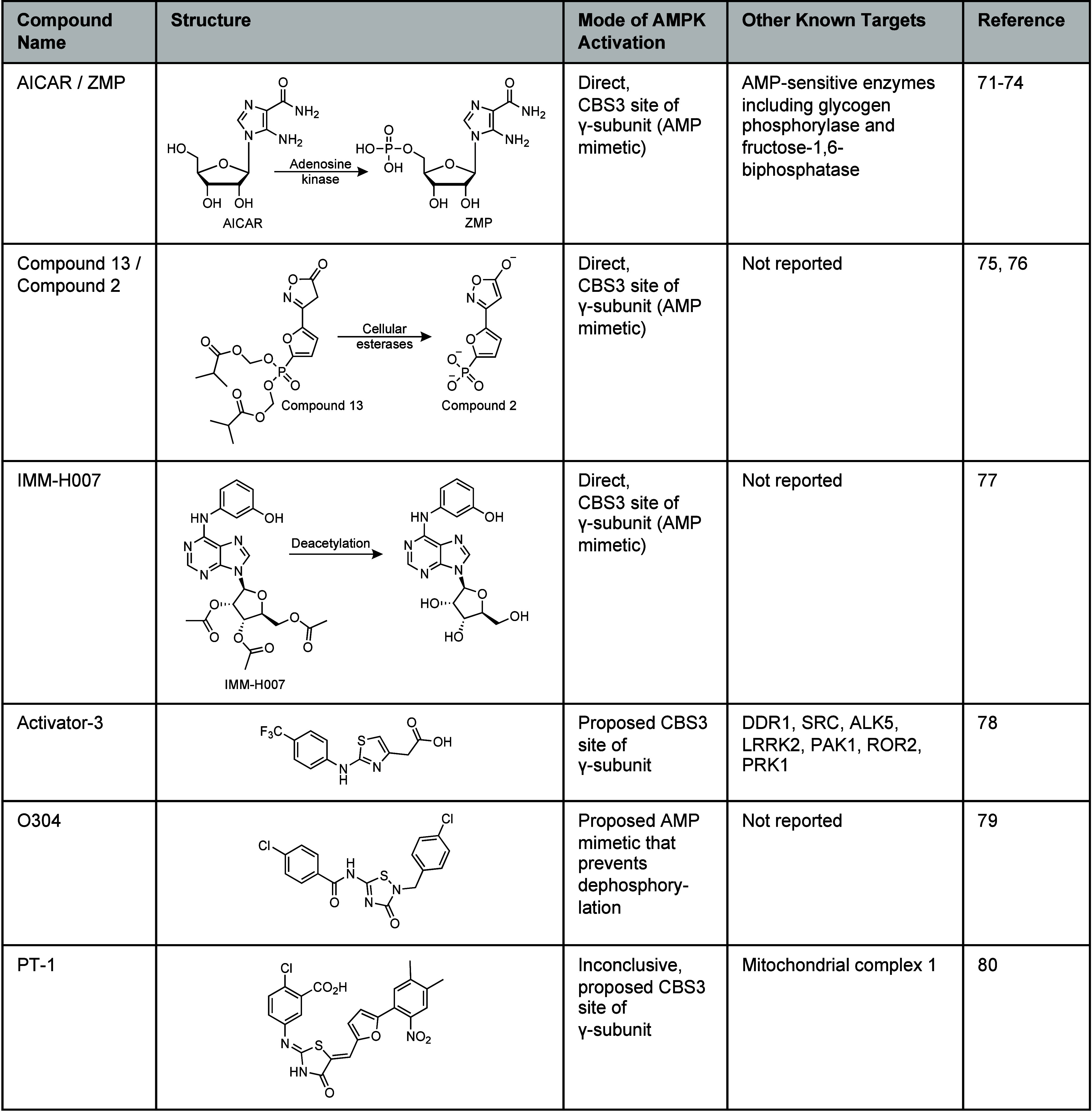
Direct AMPK Activators Acting at the CBS3 Site of the γ-Subunit^a^

a Bioactivation of AICAR, Compound 13, and IMM-H007 that are known to directly act at the CBS3 site of the γ-subunit and a list of known alternative targets. Chemical structures of Activator-3, O304, and PT-1 that are proposed to interact at the CBS3 site.

A more selective and potent AMPK activator is Compound 2 (C2) that is formed from the bioactivation of the phosphonate diester prodrug Compound 13 (C13) (Table 2).^75^ The phosphonate diester makes C13 cell-permeable and once inside the cell the prodrug can be converted by esterases to C2, a phosphonate analog of AMP. C2 is 2–3 orders of magnitude more potent than AMP and 4 orders of magnitude more potent than ZMP as an activator of AMPK.^75^ This increased potency may be due to C2 binding to the CBS sites in the γ-subunit of AMPK in a different orientation to the natural nucleotides.^76^ This alternative binding conformation may also explain why C2 does not affect other AMP-sensitive enzymes such as glycogen phosphorylase and fructose-1,6-biphosphatase.^73^ An interesting feature of C2 is that it is almost completely selective for the AMPKα1 isoform, with little or no activity with AMPKα2 and γ3 isoforms.^73^ Since the discovery of AICAR and C13, several other AMP-mimetics have been developed as AMPK activators, including the triacetyl-3-hydroxyphenyladenosine (IMM-H007) derivative of cordycepin that requires deacetylation to generate an AMP mimetic (Table 2).^77^

The thiazole, Activator-3 (Table 2), has been proposed to act as an AMP mimetic at the γ-subunit of AMPK that has been supported by mutation studies.^78^ Activator-3 can enhance AMPK phosphorylation and protects AMPK against protein phosphatase 2C (PP2C)-medaited dephosphorylation. When screened for off-target activity, DDR1, SRC, and ALK5 kinases were inhibited >50% at 10 μM, and LRRK2, PAK1, ROR2, and PRK1 were activated 30–40% at 10 μM.^78^ The thiadiazol-3-one, O304 (Table 2), was identified from a cellular screen as a pan-AMPK activator that suppresses the dephosphorylation of Thr12 in activated AMPK.^79^ The exact mechanism and site of action of O304 is unknown, but it seems to mimic the effects of AMP. A limitation of O304 is that it will only further increase pAMPK levels in cells with existing intrinsic AMPK activity.^79^ The thiazol-3-one, PT-1 (Table 2), was initially reported to activate AMPK by binding between the kinase domain and AID region of the α-subunit, a subsequent study showed that PT-1 indirectly activates AMPK by inhibiting the mitochondrial respiratory chain.^80^ However, PT-1 appeared to activate γ1 isoform AMPK complexes and did not activate γ3 isoform AMPK complexes in incubated mouse muscle, this γ-isoform selectivity could indicate interaction at the nucleotide sites.^80^ Further studies are required to support the mechanism and site of action of Activator-3 and the structurally similar O304 and PT-1 AMPK activators.

### 4.3 Allosteric Activators

Another class of AMPK activators are those that bind to sites distinct from the adenosine binding sites and use an allosteric activation mechanism.^2^ Small molecules binding to the ADaM site, a cleft located between the N-lobe of the kinase domain on the α-subunit and the β-subunit CBM, allosterically activate AMPK and protect against Thr172 dephosphorylation.^2^ In 2006, Abbott Laboratories identified the thienopyridone A-592107 (Table 3), as an activator of AMPK from a screen of ∼700,000 small molecules.^81^ Subsequent optimization led to the development of A-769662 (Table 3), as an allosteric AMPK activator that demonstrated a ∼ 50-fold improvement of AMPK activation compared with A-592107.^81,82^ Allosteric activation of AMPK with A-769662 at the ADaM site was suspected as its effect was additive with AMP,^81^ supported by mutation studies,^83^ and confirmed by cocrystallization.^10^ This ADaM site activator is only one of a few that has been tested in cancer models and was shown to promote the growth of lung and colorectal carcinoma cell lines grown under hypoxia.^59^ An analog of A-769662 was developed by GlaxoSmithKline by replacing the fused thiophene with an *N*-substituted pyrrole resulting in GSK-621 (Table 3).^84^ Although direct binding to AMPK has yet to be confirmed, GSK-621 inhibits the growth of melanoma and hepatocellular carcinoma as a single agent and in combination with lapatinib in breast cancer cells.^84−87^

**Table 3:**
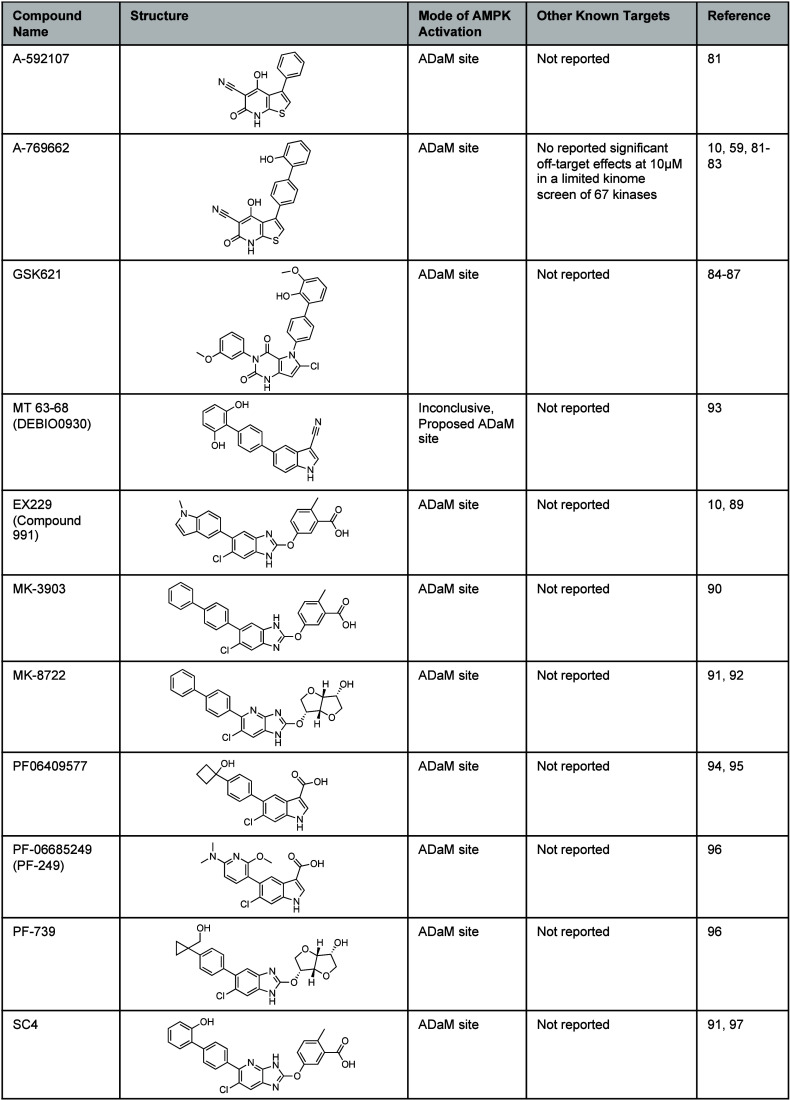
Direct AMPK Activators Acting at the AdaM Site^a^

a Chemical structures of A-592107, A-769662, GSK621, MT 63-78, EX229, MK-3903, MK-8722, PF-06409577, PF-06685249, PF-739, and SC4 and a list of known alternative targets.

A patent review in 2012 revealed that many AMPK activators under development shared structural similarity with A-769662, replacing the thienopyridone with indole, benzimidazole, and azabenzimidazole heterocyclic cores.^88^ Many of these ADaM site activators, like A-769662, tend to bind more tightly to the AMPK heterotrimer containing the β1-subunit than the β2-subunit; however, the difference in β-isoform binding was compound dependent. A potent AMPK activator EX229 (Compound 991, Table 3), identified from a high-throughput screen (HTS) by Merck demonstrated 5–10-fold greater activation of AMPK than A-769662 and exhibited a ∼ 10-fold preference for AMPKβ1.^10,89^ Subsequent optimization by a fragment library approach led to the development of MK-3903 (Table 3) that demonstrated activity against 10 of the 12 AMPK isoforms,^90^ and then MK-8722 (Table 3) as a pan-AMPK activator.^91^ The development of MK-8722 revealed important structure–activity information for pan-AMPK activation and that AMPKβ2 activation can support glucose homeostasis but this can also induce cardiac hypertrophy.^91^ Interestingly, MK-8722 was shown to inhibit pancreatic cell proliferation and migration/invasion, but these effects were found to be AMPK-independent.^92^

**Figure 4:**
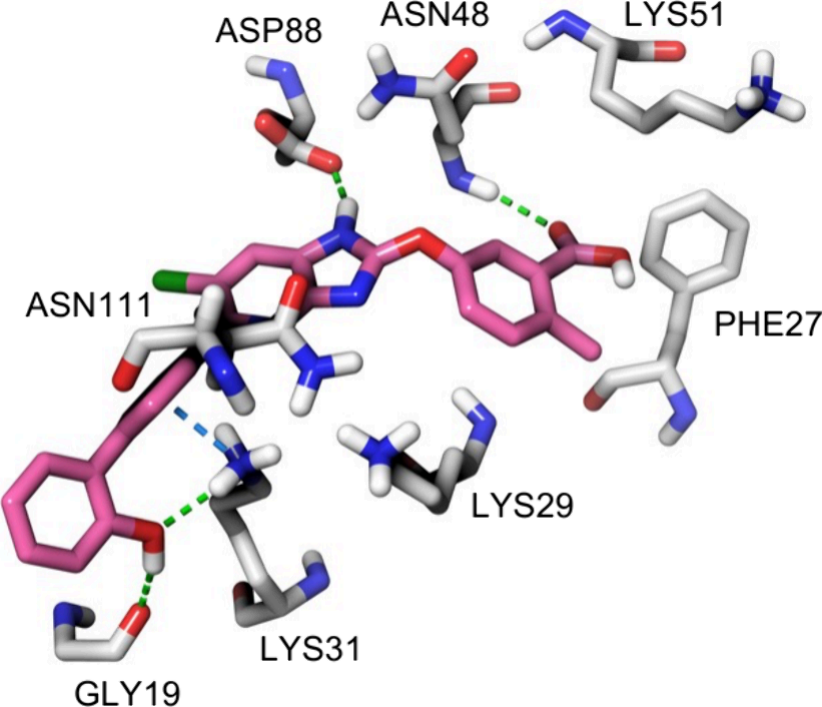
The allosteric activator SC4 docked into the ADaM site of AMPK. SC4 (carbons colored pink) docked into the ADaM site of AMPK α2β1γ1 (PDB: 4CFF).^10^ Carbons colored gray for amino acid residues. H-bonds: green dashed line. Pi-cation bonds: blue dashed line. Docking was performed using the Glide module of the Schrödinger 2024-1 Drug Discovery suite.

The indole MT 63–78 (DEBIO0930, Table 3) shows selectivity for the AMPKβ1 heterotrimers; however, binding to the ADaM site has not been conclusively demonstrated.^93^ Pfizer developed the indole PF-06409577 (Table 3), from an indazole amide HTS hit, as a β1-specific AMPK activator that advanced to clinical trial for the treatment of diabetic nephropathy; however, this trial was terminated due to rapid clearance.^94,95^ Therefore, new indole analogs were developed including the β1-specific AMPK activator PF-06685249 (PF-249, Table 3) that demonstrated increased bioavailability, prolonged half-life, and low clearance in preclinical *in vivo* studies.^96^ Pfizer also developed a series of benzimidazoles and identified PF-739 (Table 3), that is structurally similar to MK-8722, as a potent pan-AMPK activator with a slightly higher affinity for the β1-isoform.^96^ Interestingly, structure–activity studies revealed that the 4′-nitrogen of the imidazopyridine ring of MK-8722 and SC4 (Table 3) is required to facilitate a stabilizing interaction with Asp111 in AMPKβ2 isoforms (Figure 4).^91,97^

The Asn111 of the β1-isoform and Asp111 of the β2-isoform appear to be critical for modulating β-isoform targeting of AMPK activators. A distinction between MK-8722 and SC4 is that SC4 activates all six AMPKα2 and two AMPKα1 (α1β1γ1 and α1β1γ3) complexes, and it has been proposed that pan-AMPK activation of MK-8722 is due to the 2′-mannitol group interacting with conserved residues in the α-subunit. Therefore, these studies demonstrated that activation of the β2-containing AMPK isoforms could be achieved by insertion of a 4′-nitrogen in the imidazopyridine and some AMPKα isoform selectivity can be introduced by the 2′-substituent. These findings may form the foundation for the development of more isoform-selective AMPK activators, which in-turn may result in a tissue specific AMPK activator, limiting the potential for cardiac adverse events.^98^

## 5 Small Molecule AMPK Inhibitors

There are few known, potent and selective small molecule AMPK inhibitors. At present, all the reported AMPK inhibitors target the ATP-binding site of the catalytic α-subunit of AMPK. In this section we will summarize the commonly used Compound C and several emergent AMPK inhibitors (Table 4).

**Table 4:**
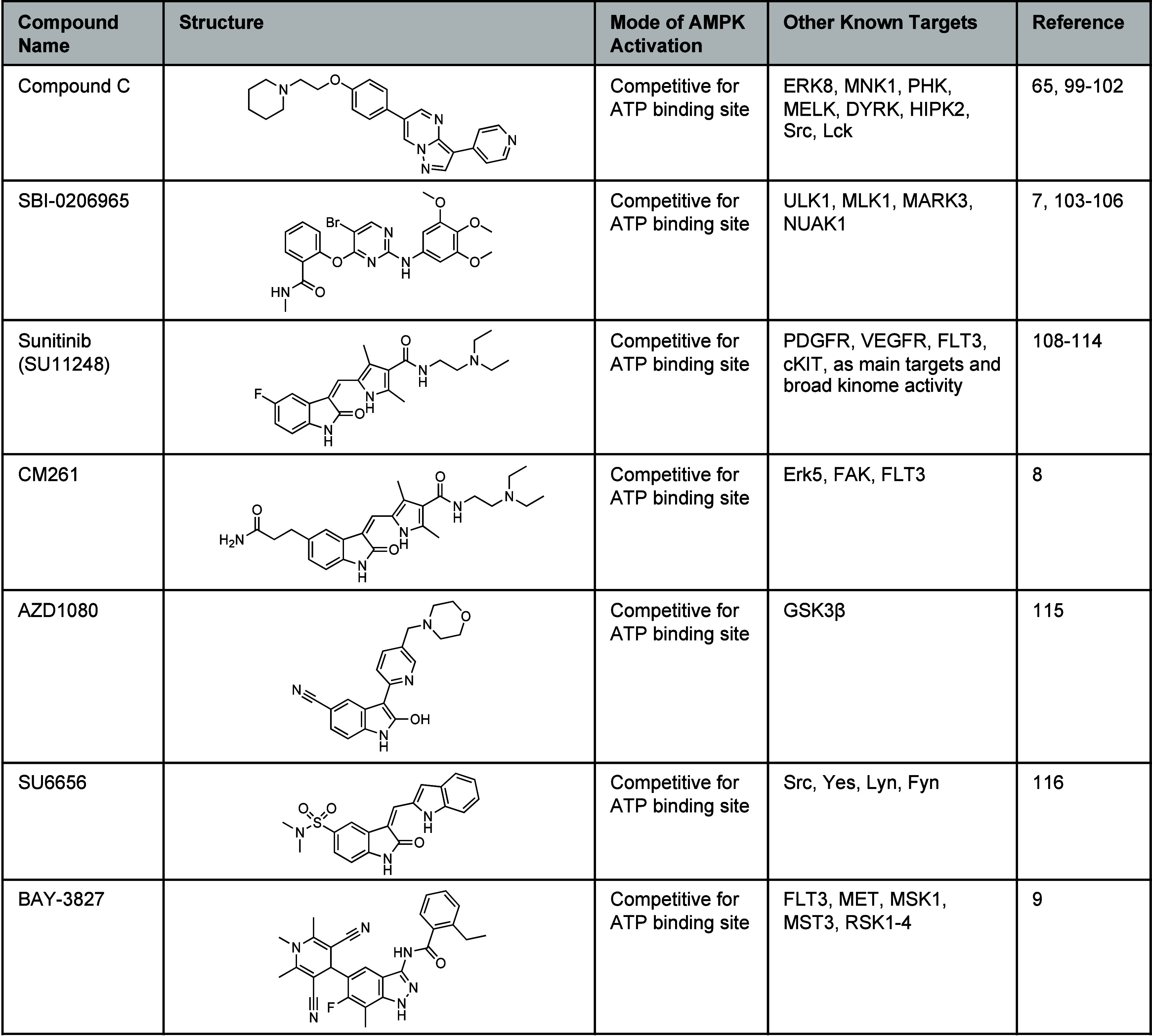
Direct AMPK Inhibitors Acting at the ATP-Binding Site of the α-Subunit^a^

a Chemical structures of compound C, SBI-0206965, sunitinib, CM261, AZD1080, SU6656, and BAY-3827 that directly act at the catalytic ATP-binding site and a list of known alternative targets.

### 5.1 ATP Competitive Inhibitor Compound C

The pyrazolopyrimidine compound C (dorsomorphin; Table 4) was identified as an AMPK inhibitor from a HTS and has been widely used as an ATP-competitive AMPK inhibitor in biochemical, cell-based, and *in vivo* assays.^65,99^ Despite widespread use and a K_i_ of 109 nM from an *in vitro* [^33^P]-ATP kinase activity assay, micromolar concentrations ∼40 μM of compound C are required for inhibition of cellular AMPK activty.^65,99^ Compound C also exhibits broad-spectrum kinome activity and inhibits a number of other kinases more potently than AMPK, including ERK8, MNK1, PHK, MELK, DYRK, HIPK2, Src, Lck.^100^ In addition, several studies have reported that compound C disrupts various biological events independently of AMPK inhibition, and that its anticancer effects are AMPK independent.^101,102^ The high micromolar concentrations of compound C required for intracellular AMPK inhibition exacerbates its off-target effects, confers potent cytotoxicity, and it is unclear what biological effects are due to AMPK inhibition. Therefore, compound C has little use or scope for development as a selective AMPK inhibitor.

### 5.2 Type II Inhibition by SBI-0206965

An active site competitive kinase screen identified the 2-aminopyrimidine, SBI-0206965 (Table 4), as a potent and selective inhibitor of the autophagy initiator ULK1; however, SBI-0206965 also displayed activity against AMPKα1 and α2 complexes.^103^ Further investigation revealed that SBI-0206965 was a more potent AMPK inhibitor than compound C in an *in vitro* [^32^P]-ATP kinase activity assay (AMPKα1 IC_50_ 0.40 μM versus 15.89 μM).^7^ A cocrystal structure of SBI-0206965 in the α2-subunit of AMPK showed that SBI-0206965 overlaps with the ATP-binding site with the DFG in an open conformation and kinetic studies supported that SBI-0206965 inhibits AMPK with type II inhibitor characteristics.^7^ A kinome screen against 50 kinases (9% of the human kinome) using an *in vitro* [^33^P]-ATP kinase activity assay reported that SBI-0206965 (0.25 μM) was more selective than compound C (2.5 μM); however, a 10-fold higher concentration of compound C was used in the screen favoring selectivity for SBI-0206965.^7^ A more recent kinome screen against 140 kinases (26% of the kinome) using an *in vitro* [^33^P]-ATP kinase activity assay demonstrated that SBI-0206965 inhibits several kinases, including MLK1, MARK3, and NUAK1, equally or more potently than AMPK or ULK1.^104^ Micromolar concentrations >10 μM of SBI-0206965 are required for inhibition of cellular AMPK activity determined by measuring pACC expression.^7,104^ However, SBI-0206965 at 1–10 μM has been shown to reduce cell viability by 50% and initiate apoptosis in several cell lines; therefore, reductions in pACC expression could be a result of reduced cell population rather than AMPK inhibition.^105,106^ The pharmacokinetics and metabolism of SBI-0206965 has been evaluated in rodent models as a potential treatment for GBM; however, it is unlikely that SBI-0206965 will transition to clinical evaluation as it demonstrated poor absorption and rapid first-pass hepatic metabolism.^107^

### 5.3 ATP Competitive Inhibition by Oxindoles

The multikinase inhibitor sunitinib (SU11248, Table 4) is FDA approved for treating renal cell carcinoma and imatinib-resistant gastrointestinal stromal tumors.^108^ Sunitinib was originally designed as an inhibitor of receptor tyrosine kinases, with receptors for platelet-derived growth factor (PDGFR) and vascular endothelial growth factor (VEGFR) being its main targets.^109,110^ Sunitinib has broad-spectrum activity across the kinome and is also a potent ATP-competitive AMPK inhibitor with an IC_50_ of 0.045 μM, compared with an IC_50_ of 2.38 μM for compound C in the same *in vitro* time-resolved fluorescence resonance energy transfer (TR-FRET) AMPK kinase activity assay.^111^ Although sunitinib inhibits both AMPKα1 and α2 isoforms, it seems to exhibit some selectivity for AMPKα1 over AMPKα2 in a competitive binding assay (K_d_ 19 nM versus K_d_ 89 nM),^112^ and in four kinase activity assays (IC_50_ 6.7–37 nM versus IC_50_ 4.8–72 nM).^113^ The clinical cardiotoxicity of sunitinib has been attributed to the inhibition of AMPK and 90 kDa ribosomal S6 kinase (RSK) kinases, which may limit clinical development of AMPK inhibitors.^113,114^

**Figure 5:**
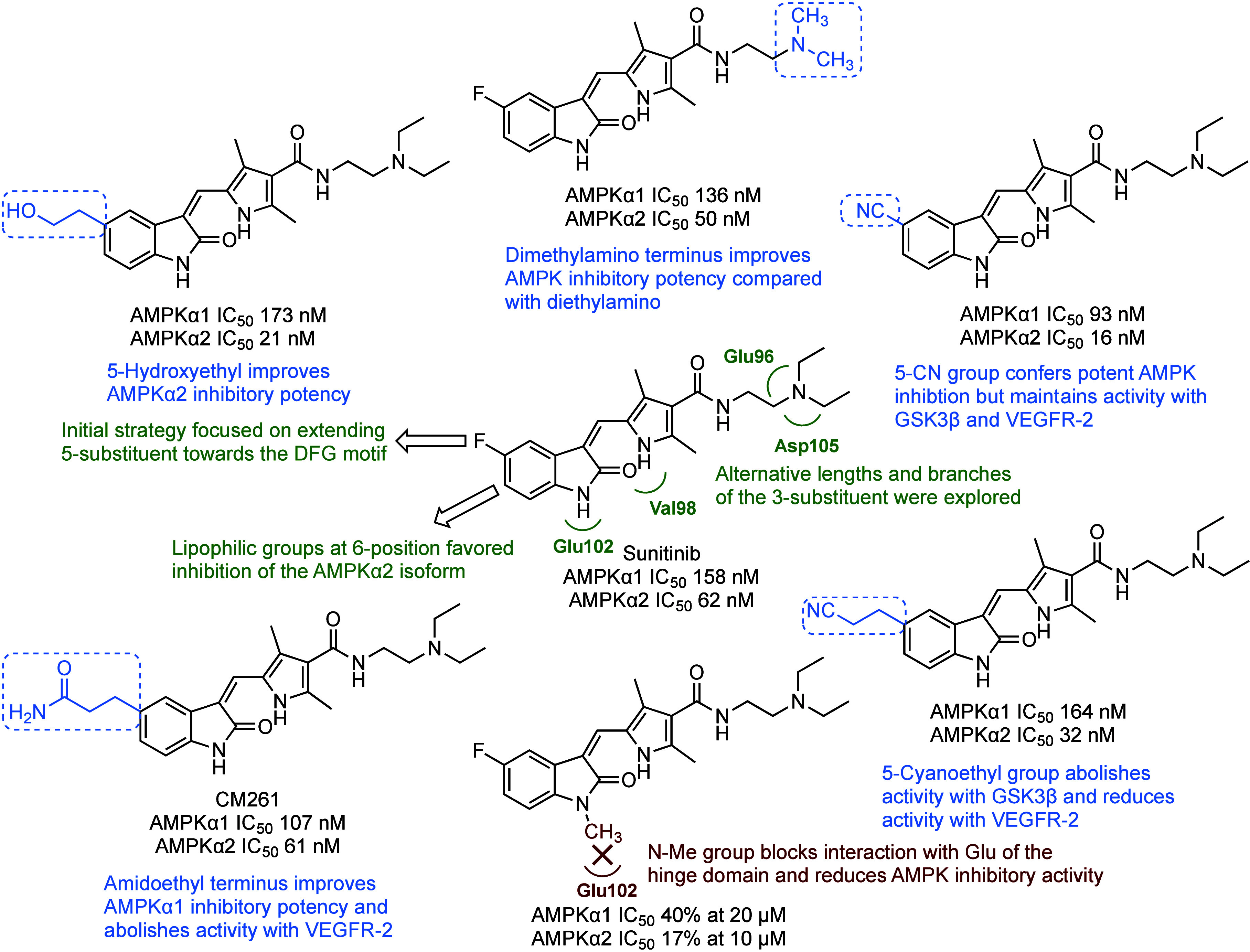
Optimization of sunitinib to improve AMPK inhibition and selectivity. IC_50_ values were derived from a TR-FRET kinase activity assay.^8^

A structure–activity study performed by Matheson et al., evaluated 25 sunitinib analogs as AMPK inhibitors and found that 5-substituted oxindoles, designed to interact with the DFG of the catalytic ATP-binding site, could improve inhibition of AMPKα1 or AMPKα2 activity (Figure 5).^8^

The oxindole CM261 emerged as the lead AMPK inhibitor from this study with improved AMPKα1 inhibition over sunitinib in a TR-FRET kinase activity assay (IC_50_ 107 nM versus IC_50_ 158 nM, Table 4). The cellular target engagement of AMPK by these oxindoles was quantitatively measured by ELISA, and a 50% decrease of pACC levels was observed for several oxindoles at 5 μM in the K562 chronic myeloid leukemia cell line. Interestingly, many of these oxindoles did not exhibit the potent cytotoxicity of sunitinib in K562 cells, and this could be due to improved kinase selectivity. In a kinome screen containing over 400 kinases (77% of the kinome), CM261 showed reduced activity against kinases in the receptor tyrosine kinase (RTK) family, which are common target kinases for sunitinib. This structure–activity study demonstrated that side-chain modifications around the oxindole core could improve AMPK inhibitory potency and selectivity.^8^

Another oxindole, AZD1080 (Table 4), a potent inhibitor of glycogen synthase kinase-3β (GSK3β), was developed for the treatment of Alzheimer’s disease, but abandoned due to nephrotoxicity in Phase I trials.^115^ However, in a limited kinome profile against 24 kinases (5% of the kinome), AZD1080 at 10 μM inhibited the kinase activity of only GSK3β and AMPK by more than 50% in an *in vitro* [^33^P]-ATP kinase assay.^115^ While AZD1080 is not a potent inhibitor of AMPK, the limited kinome screen provides further support that side-chain modification around the oxindole ring has the potential to introduce AMPK selectivity.

Interestingly, the oxindole-based Src kinase inhibitor SU6656 (Table 4), also acts as an ATP-competitive inhibitor of AMPK; however, this oxindole can paradoxically activate AMPK.^116^ This paradoxical activation is due to the binding of SU6656 at the catalytic site inducing a conformational change in the activation loop, promoting LKB1-mediated Thr172 phosphorylation and a further conformation change resulting in the dissociation of the inhibitor and phosphorylation of downstream AMPK targets.^116^ This mechanism of paradoxical activation of AMPK may not be unique to SU6656.

### 5.4 BAY-3827: a Potent and Selective AMPK Inhibitor

**Figure 6:**
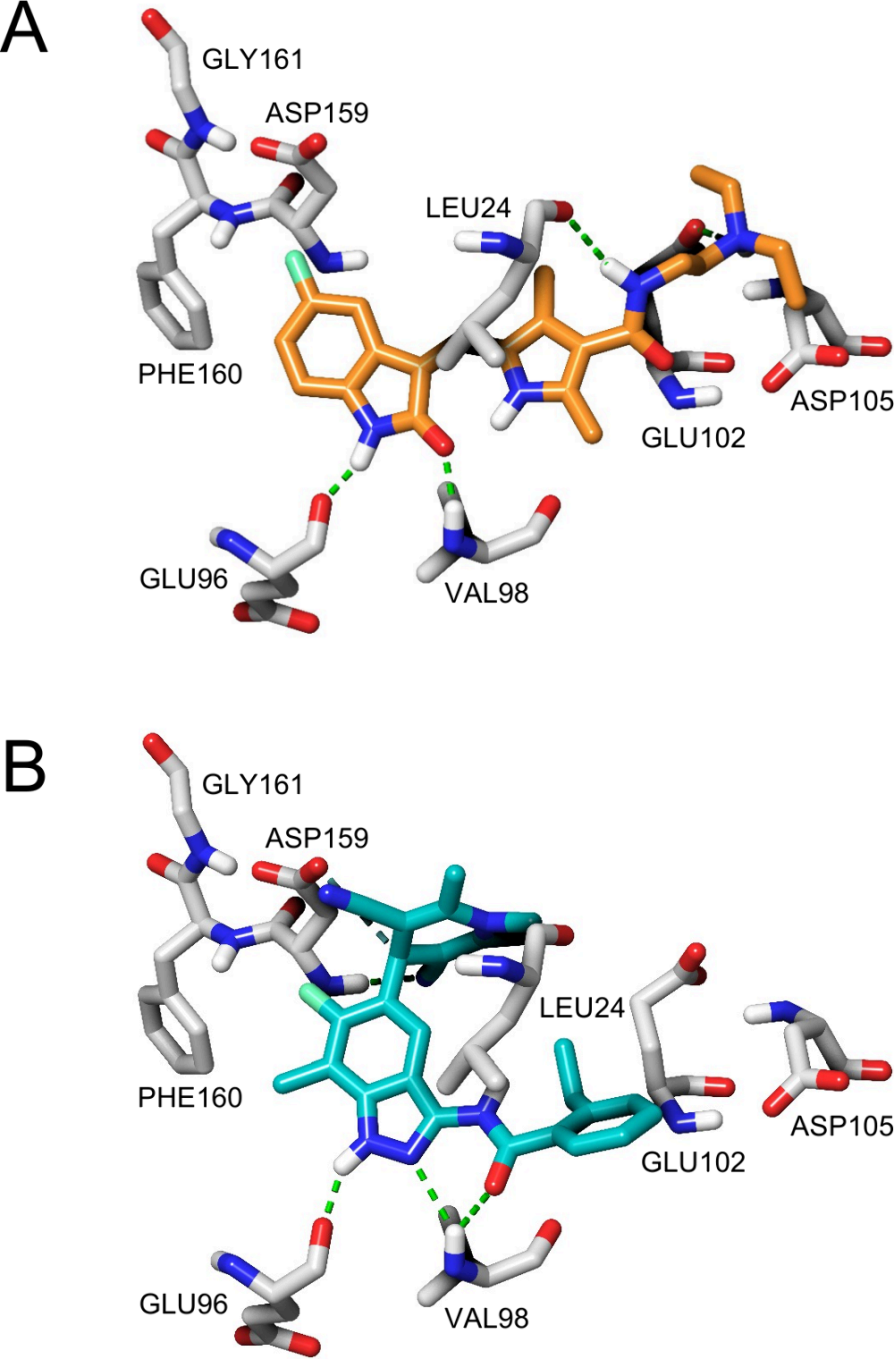
AMPK Inhibitors docked into the ATP-binding site of AMPK. A) Sunitinib (carbons colored orange) and B) BAY-3827 (carbons colored cyan) docked into the catalytic ATP-binding site of AMPK α1β2γ1 (PDB: 4REW).^117^ Carbons colored gray for amino acid residues. H-bonds: green dashed line. Pi-cation bonds: blue. Salt bridges: purple. Docking was performed using the Glide module of the Schrödinger 2024-1 Drug Discovery suite.

Recently, Bayer AG reported the selective and potent AMPK inhibitor BAY-3827 (Table 4).^9^ A HTS of ∼4 million compounds was performed to identify AMPK inhibitors and identified a dihydropyridine-dicarbonitrile-based compound, that was further optimized resulting in the indazole BAY-3827.^9^ In this study it was reported that BAY-3827 inhibits AMPKα2β1γ1 kinase activity in a TR-FRET assay with IC_50_ values of 1.4 nM at low 10 μM ATP, and 15 nM at high 2 mM ATP concentrations with 2 μM AMP under both conditions. A homogeneous time-resolved fluorescence (HTRF) assay was used to determine cellular AMPK inhibition by measuring pACC1 levels in cell lysates of the LNCaP and VCaP prostate (IC_50_ ∼ 100 nM), IMR-32 neuroblastoma (IC_50_ ∼ 150 nM), and Colo320 colon adenocarcinoma (IC_50_ ∼ 400 nM) cell lines.^9^ The antiproliferative effect of BAY-3827 varied in a panel of prostate cancer cell lines, which was shown to correlate with androgen-dependent prostate cancer cell lines and multiple myeloma cell lines.^9^ A kinome profile against 331 kinases (64% of the kinome) revealed that BAY-3827 was reasonably selective with activity (>80% inhibition) against AMPKα1, AMPKα2, FLT3, MET, MSK1, MST3, and RSKs1–4; however, there were notable omissions as the screening concentration of BAY-3827 was not provided and kinases such as ERK, KIT, and VEGFR-2 were not included in the screen.^9^ There were some additional observations from the kinome screen in that BAY-3827 did not display AMPK isoform selectivity and demonstrated potent activity against some members of the AGC cytoplasmic serine/threonine kinase family, including all the RSK isoforms. This could be important as although sunitinib and BAY-3827 are structurally distinct and have different binding conformations in the catalytic ATP-binding site of AMPK (Figure 6), they potently inhibit the AMPK and RSK kinases that are considered to cause the cardiotoxicity of sunitinib.^113,114^

A recent study found that SBI-0206965 and BAY-3827 promote Thr172 phosphorylation, with SBI-0206865 promoting LKB1-mediated phosphorylation and BAY-3827 protecting Thr172 dephosphorylation and this may be another example of paradoxical activation of AMPK.^118^ In addition, BAY-3827 demonstrated poor bioavailability that may limit its use as a therapeutic, but given its potency and high level of selectivity it should now be considered as a standard for AMPK inhibition studies and as a chemical tool for exploring the role of AMPK in cell systems.^118^

## 6 Conclusions and Future Perspectives

Small molecules have been shown to interact with three distinct binding sites of AMPK to modulate catalytic activity: the ATP-binding site of the catalytic α-subunit, the ADaM site, and the adenine nucleotide binding sites of the γ-subunit. Although the target binding sites for many of these small molecules have been resolved by X-ray crystallography, there is complexity in the determination of which site small molecules bind and how they impact AMPK activity. Small molecule adenosine analogs that target the γ-subunit have the potential to inhibit as well as promote AMPK activity, and there are increasing cases of paradoxical activation by small molecule inhibitors at the ATP-binding site of the catalytic α-subunit.^116^ In cell systems the effects of small molecules on AMPK activity are often monitored by measuring the levels of pACC by Western blot or ELISA; however, these are not direct measures of target engagement. The effects of these small molecules on pACC may be due to upstream interactions, influence on noncanonical AMPK activation pathways, or other off-target effects, rather than direct modulation of cellular AMPK activity. Therefore, when using these determination methods in cancer cell systems it would be difficult to attribute the anticancer effect of small molecule modulators to AMPK activity alone. Methods used to induce AMPK expression and activation in cells such as pretreating cells with the glycolysis inhibitor 2-deoxy-d-glucose (2-DG) or culturing in low glucose media or hypoxic conditions,^8,119^ may activate many other signaling networks. Therefore, when using these approaches to activate AMPK to determine the effectiveness of AMPK modulators in cancer cell models this may lead to confounding results as the impact on cell viability may be due to broader events than targeting AMPK activity.^8^ Overall, for the development of small molecule AMPK modulators as anticancer agents there needs to be a more rigorous evaluation of target engagement.

The isoform selectivity of small molecule AMPK modulators has the potential to play a key role in cancer treatment, where patterns of AMPK isoforms are expressed in different cancer types. A central focus of the ADaM site activators has been to target AMPK isoforms, primarily the AMPKα2β2γ3 complex of skeletal muscle to facilitate glucose uptake and reduce blood glucose.^2^ There has been some notable progress with this endeavor as evidenced by SC4, that activates AMPKα2β2 complexes.^97^ However, some of these AMPK activators, such as the pan-AMPK activator MK-8722, have displayed cardiac hypertrophy in animal models, presumably as a result of potentiating AMPKα2 activity.^2^ Interestingly, the cardiotoxicity of the multikinase inhibitor sunitinib has been attributed in-part to AMPKα2 inhibition.^113^ From an analysis of *STK11*, *PRKAA1* and *PRKAA2* genes in human cancers and normal tissue in the cBioPortal database, it was noted that *STK11* the gene for LKB1 was often mutated in cancer, the *PKAA1* gene encoding AMPKα1 was frequently amplified suggesting a tumor promoter function, whereas the *PKAA2* gene encoding AMPKα2 was often mutated suggesting a tumor suppressor function.^37^ This suggests that inhibitors that target the AMPKα1 isoform may be more effective anticancer agents with reduced cardiotoxicity; however, although our studies and those of others further support that AMPKα1 is the predominant isoform in cancer that supports cell survival,^8,60^ the presence of AMPKα2 in other cancers cannot be dismissed.^6^

It may be a significant challenge to develop small molecule AMPK inhibitors that are selective for specific AMPK isoforms due to the α-subunit homology around the ATP-binding site; however, AMPK inhibitors that are tumor-selective rather than systemically inhibiting AMPK may be more clinically useful with reduced side-effects. Therefore, as an anticancer strategy it may be advantageous to develop hypoxia-activated prodrugs (HAPs) of AMPK inhibitors^120^ that would be more tumor-selective than AMPK isoform-selective and may reduce systemic effects and toxicities. In this approach, AMPK inhibitors would be bioactivated and concentrated in regions of tumor hypoxia (not in the oxygenated tissue of the heart) where cancer cells and the drug-resistant CSCs may be more sensitive to AMPK inhibition. This would negate the need for isoform-selective AMPK inhibitors as it would incorporate a tier of tumor-selectivity and may reduce systemic effects and toxicities. In cancer, AMPK activation and subsequent metabolic reprogramming is becoming a recognized mechanism for drug resistance,^64,121,122^ while AMPK inhibition may be sufficient to eradicate cancer cells that are sensitive to changes in the cellular AMP:ATP ratio, but it is more likely that AMPK inhibition would chemosensitize cancer cells and rational synergistic combination therapies will have to be identified for successful treatment.

## Abbreviations Used

2-DG: 2-deoxy-d-glucose
ACC: acetyl coenzyme A carboxylase
ADaM: allosteric drug and metabolite
AICAR: 5-aminoimidazole-4-carboxamide-1-β-d-ribofuranoside
AID: autoinhibitory domain
AKAP1: A kinase anchor protein
ALK5: activin receptor-like kinase-5
AMPK: AMP-activated protein kinase
CaMKK2: Ca^2+^/calmodulin dependent protein kinase 2
CBM: carbohydrate-binding module CBS, cystathionine β-synthase
CREB1: cAMP response element binding protein-1
CSC: cancer stem cell
DDR1: discoidin domain receptor tyrosine kinase 1
DYRK: dual-specificity tyrosine-regulated kinase
EF2K: elongation factor 2 kinase
ERK8: extracellular signal-regulated kinase 8
FA: fatty acid
FAO: fatty acid oxidation
FLT3: FMS-related receptor tyrosine kinase 3
GBM: glioblastoma multiforme
GLUT: glucose transporter
GSC: glioblastoma stem-like cells
GSK3β: glycogen synthase kinase-3β
HAP: hypoxia-activated prodrug
hERG: human ether-a-go-go-related gene
HIF-1α: hypoxia inducible factor 1α
HIPK2: homeodomain-interacting protein kinase 2
HMGR: 3-hydroxy-3-methylglutaryl-CoA reductase
HSC: hematopoietic stem cells
HTRF: homogeneous time-resolved fluorescence
IMP: inosine monophosphate
Lck: Lymphocyte-specific kinase
LKB1: liver kinase B1
LRRK2: leucine-rich repeat kinase 2
LSC: leukemic stem cell
MARK3: microtubule affinity-regulating kinase 3
MELK: maternal embryonic leucine zipper kinase
MET: mesenchymal-epithelial transition factor
MLK1: mixed lineage kinase 1
MNK1: MAP kinase-interacting kinase 1
MSK1: mitogen- and stress-activated protein kinase 1
MST3: mammalian STE20-like protein kinase 3
mTORC: mammalian target of rapamycin complex
NRF2: nuclear factor erythroid 2-related factor 2
NUAK1: NUAK family SNF1-like kinase 1
PAK1: P21 (RAC1) activated kinase 1
PDGFR: platelet-derived growth factor
PD-L1: programmed cell death ligand 1
PFK1: phosphofructokinase 1
PFKFB3: 6-phosphofructo-2-kinase/fructose-2,6-bisphosphatase 3
PHK: phosphorylase kinase
PI3K: phosphoinositide 3 kinase
PP2C: protein phosphatase 2C
PRN1: serine/threonine-protein kinase N1
PTEN: phosphatase and tensin homologue
ROR2: receptor tyrosine kinase-like orphan receptor 2
ROS: reactive oxygen species
RSK: 90 kDa ribosomal S6 kinase
RTK: receptor tyrosine kinase
TAK1: TGF-β activating kinase 1
T-ALL: T-cell acute lymphoblastic leukemia/lymphoma
TBC1D1: TBC1 Domain Family Member 1
TME: tumor microenvironment
TOR: target of rapamycin
TSC2: tuberous sclerosis complex 2
TXNIP13: thioredoxin-interacting protein
ULK1: unc-51-like kinase 1
VEGFR: vascular endothelial growth factor receptor
YAP: yes-associated protein
